# Rapid Changes in Cortical and Subcortical Brain Regions after Early Bilateral Enucleation in the Mouse

**DOI:** 10.1371/journal.pone.0140391

**Published:** 2015-10-09

**Authors:** Olga O. Kozanian, Charles W. Abbott, Kelly J. Huffman

**Affiliations:** 1 Department of Psychology, University of California Riverside, 900 University Avenue, Riverside, CA 92521, United States of America; 2 Interdepartmental Neuroscience Program, University of California Riverside 900 University Avenue, Riverside, CA 92521, United States of America; Rutgers University, UNITED STATES

## Abstract

Functional sensory and motor areas in the developing mammalian neocortex are formed through a complex interaction of cortically intrinsic mechanisms, such as gene expression, and cortically extrinsic mechanisms such as those mediated by thalamic input from the senses. Both intrinsic and extrinsic mechanisms are believed to be involved in cortical patterning and the establishment of areal boundaries in early development; however, the nature of the interaction between intrinsic and extrinsic processes is not well understood. In a previous study, we used a perinatal bilateral enucleation mouse model to test some aspects of this interaction by reweighting sensory input to the developing cortex. Visual deprivation at birth resulted in a shift of intraneocortical connections (INCs) that aligned with ectopic *ephrin A5* expression in the same location ten days later at postnatal day (P) 10. A prevailing question remained: Does visual deprivation *first* induce a change in gene expression, followed by a shift in INCs, or vice versa? In the present study, we address this question by investigating the neuroanatomy and patterns of gene expression in post-natal day (P) 1 and 4 mice following bilateral enucleation at birth. Our results demonstrate a rapid reduction in dorsal lateral geniculate nucleus (dLGN) size and *ephrin A5* gene expression 24-hours post-enucleation, with more profound effects apparent at P4. The reduced nuclear size and diminished gene expression mirrors subtle changes in *ephrin A5* expression evident in P1 and P4 enucleated neocortex, 11 and 8 days prior to natural eye opening, respectively. Somatosensory and visual INCs were indistinguishable between P1 and P4 mice bilaterally enucleated at birth, indicating that perinatal bilateral enucleation initiates a rapid change in gene expression (within one day) followed by an alteration of sensory INCs later on (second postnatal week). With these results, we gain a deeper understanding of how gene expression and sensory input together regulate cortical arealization and plasticity during early development.

## Introduction

The mammalian neocortex is a complex structure in the central nervous system that contains a unique set of functional and anatomical properties. This structure is responsible for integration of naturally occurring cognitive demands in the everyday environment, including language, decision-making, motivation and sensori-motor processing. During development, sensory and motor areas are formed in the neocortex through a process called arealization. The mechanisms responsible for cortical area development, or arealization, have been debated for years, with two contradictory models initially emerging: the Protomap and Protocortex hypotheses [1–2]. In this paper, we begin to unravel ways in which these two hypothetical mechanisms interact within the developing visual system of mice.

### Early visual system development

During early development, visual cortical map formation is driven by spontaneous retinal activity where retinal ganglion cells create excitatory potentials that propagate across the retina in the form of ‘waves’ [3]. Specifically, cholinergic retinal waves are essential during the first postnatal week when retinotopic maps are being established and refined in the dorsal lateral geniculate nucleus of the thalamus (dLGN) [4] and visual cortex [5], before cholinergic independent glutamatergic waves appear in the second postnatal week [6]. Spontaneous retinal activity and cortical gene expression occur together in early development, during embryogenesis. That is, spontaneous neural activity coupled with a combination of Eph-A-ephrin-A signaling guide the formation of visual maps [7]. The Eph family of receptor tyrosine kinases and their cell-surface bound ligands, the ephrins, contribute greatly to thalamocortical axon patterning. Eph receptors and ephrins play an important role during early neural development by acting as markers for topographic map establishment by triggering contact-mediated repulsion or attraction [8–12]. A role for EphA/ephrin-A interactions in thalamocortical patterning has been inferred from their normal graded expression patterns in the developing thalamus and cortex, and studies showing defects in ephrin-A knockout mice [13–14].

An important feature of cortical arealization, or cortical patterning, is the establishment of precise intraneocortical connections (INCs), which serve as the primary network for cortical function. Neocortical gene expression is believed to drive initial arealization and targeting of INCs prior to eye opening, during preliminary developmental stages [15–19]. Subsequent sensory experience after eye opening is hypothesized to refine visual cortex development after the initial patterning [20]. We hypothesize that both activity-independent, cortically intrinsic mechanisms, such as gene expression, and activity-dependent mechanisms, such as spontaneous retinal wave activity, act in tandem to pattern visual subcortical and cortical regions. To test this hypothesis, we created a visual deprivation model in an attempt to experimentally manipulate sensory input and conduct anatomical and molecular analyses to uncover the extent in which sensory input impacts the developmental patterning of the system. Results from our previous work demonstrated that removal of all visual input to the neocortex at birth, via bilateral enucleation, resulted in short-term plasticity as early as postnatal (P) day 10, prior to eye opening. At this point, correlated ectopic INCs and ectopic *ephrin A5* expression were observed at the somatosensory-visual (S-V) area border [21]. Thus, by ten days post enucleation, both gene expression and INCs were disrupted. As cortical gene expression has been previously linked to INC development [16] we extended this research to study younger ages in an attempt to determine the order of visual-deprivation induced events and uncover how quickly these molecular and neuroanatomical changes can occur after bilateral enucleation in the newborn. Specifically, in this paper, we show the effects of newborn bilateral enucleation on cortical and subcortical anatomy and *ephrin A5* expression during the first postnatal week (i.e., P1 and P4).

## Methods

### Mouse colony

All studies were conducted under a research protocol approved by the Institutional Animal Care and Use Committee at the University of California, Riverside. All experimental and breeding mice were of CD1 background, originally purchased from Charles River Laboratories International, Inc., Wilmington, Massachusetts, USA.

### Newborn bilateral enucleation

Enucleation procedures were conducted just after birth (P0). A maximum of 1 hour of nursing was allowed prior to surgery. Pups were anesthetized with a mixture of ketamine (40 mg/kg, IP) and xylazine (5 mg/kg, IP), then placed on ice for 1 to 4 minutes prior to bilateral eye removal [21–22]. A toe-pinch was performed to confirm appropriate level of surgical anesthesia, after which the eyelid was cleaned using aseptic techniques. Next, an incision was made in the eyelid with a scalpel, the eye lifted away from the orbit with forceps, and separated from the optic nerve with surgical scissors. Subsequently, the eyelid was sealed with 0.5μl to 1μl of tissue adhesive (Surgi-Lock instant liquid tissue adhesive, Fisher Scientific, Pittsburgh, PA, USA). The procedure was then replicated on the other eye. After enucleation, the pups were immersed in lukewarm water bath for 30 seconds followed by an application of Lidocaine hydrochloride jelly USP, 2% (Akorn, Lake Forest, IL, USA and erythromycin ophthalmic ointment USP, 0.5% (Bausch & Lomb, Rochester, NY, USA) to prevent pain, swelling and infection. Pups were housed with their mother in a cage with nesting material before and after surgery. Control mice were treated as sham and anesthetized; they were recovered without subsequent surgical procedures. All experimental and sham control mice were housed with their mother under normal animal-facility illumination (12-hour light/dark cycle).

### Tissue preparation

Experimental animals were subjected to an early sensory deprivation period (either 1 or 4 days), after which both enucleated and control mice were given a lethal dose of sodium pentobarbital (100mg/kg) and transcardially perfused with 4% paraformaldehyde (PFA) in 0.1M phosphate buffer (pH 7.4). Brains were removed from the skull, post-fixed for a 24-hour period and hemisected. One hemisphere of each brain was used for postmortem tracing and thalamic measures, and the opposite hemisphere used for *in situ* RNA hybridization experiments. Following dye crystal placement, hemispheres used for postmortem dye tracing were stored in 4% PFA at room temperature during the 6–10 weeks required for tracer transport. Hemispheres reserved for *in situ* RNA hybridization were dehydrated in methanol, and stored at -20°C until further processing.

### Gene expression assays

Standard protocols and methods for non-radioactive free-floating *in situ* RNA hybridization were used to visualize gene expression. *Ephrin A5* (gift from John Rubenstein, UCSF) probe was used to identify the patterns of neocortical and dLGN gene expression at P1 and P4, in experimental and control murine offspring. Previously dehydrated hemispheres reserved for hybridization were rehydrated, embedded in gelatin-albumin and sectioned in the coronal plane at 100μm using a Vibratome. All sections were mounted in glycerol onto glass slides, cover slipped and photographed as described below following hybridization. A minimum of six replicate hemispheres for each age and condition was required for gene expression assays.

### Anatomical tracing techniques and DAPI staining

To determine patterns of ipsilateral INC development in control and bilaterally enucleated P1 and P4 mice, single crystals of DiI and DiA (Invitrogen, San Diego, CA, USA) were placed in two discrete dye placement locations (DPLs) after overnight post-fixation with 4% PFA at -20°C: the parietal (putative somatosensory cortex) and occipital (putative visual cortex) lobes of postmortem neocortical tissue. Methods for dye crystal placement have been described in detail elsewhere [21]. A dye placement grid was used to place each dye crystal in a morphologically defined location; this improved the reliability of the crystal placement. Following dye placement, brains were re-immersed in 4% PFA and stored at room temperature for 6–10 weeks to allow for transport of the tracer after dye placement. The medial side of the hemisected-injected brain was examined under a fluorescent dissection microscope prior to sectioning to confirm the transport of dye to the thalamus; internal capsule and thalamic labeling observed through the near-translucent tissue was indicative of the retrograded tracer reaching the thalamic nuclei. We use this method to optimize our dye transport times. All tissue was embedded in 5% low melting point agarose and sectioned in the coronal plane at 100μm using a Vibratome. Sections were immediately counter-stained with crystallized 4’,6-diamidine-2-phenylindole dihydrochloride (DAPI; Roche, Nutley, NJ, USA), mounted onto glass slides, coverslipped using Vectashield mounting medium for fluorescence (Vector Laboratories, Inc., Burlingame, CA, USA) and digitally imaged as described below. A minimum of six replicate hemispheres for each age and condition were required for INC tracing and DAPI staining.

### Analysis of gene expression

All sections processed for *in situ* RNA hybridization were imaged under a bright field scope with a digital high resolution Zeiss Axio camera using Axiovision software (Version 4.7) coupled to a Zeiss Stereo Discovery V12 stereomicroscope. ISH sections were analyzed both qualitatively and quantitatively through transcript density measures. Specifically, transcript density of *ephrin A5* was quantified using ImageJ software. Digital images were first converted to a binary form with a distinct threshold being sustained across identical anatomical levels in all replicates (n = 6 control and n = 6 enucleates). Laminar boundaries for quantifying regions of interest (ROIs) were distinguished by identifying landmarks using DAPI staining that co-registered with *in situ* hybridization of *ephrin A5*, thus allowing us to adequately define ROIs in superficial, middle and deep cortical layers. ROIs were then defined by electronically positioning grids over static position in P1 and P4 cortex of control and enucleated mice. Individual measures of each area were taken to determine transcript density in the specific region. The presence of transcript signal was measured, and reported as area fraction of total ROI.

Values for ISH density are reported as the mean ± standard error. The mean for each control group and enucleated group was established from the 6 measurements in control tissue, and 6 measurements in enucleated tissue. Two-sample independent t-tests were conducted to compare gene expression density in the ROI across control and enucleated brains. A *P*-value of less than 0.05 was chosen for statistical significance between groups.

Thalamic nuclear (dLGN) area of *ephrin A5* expression was quantified in serial sections at a fixed magnification using ImageJ. Borders of the dLGN were drawn using ImageJ at a fixed magnification, with experimenter being blind to the condition when defining the borders of the nucleus. Borders were determined as follows: the ventral lateral border of the dLGN was marked by the intergeniculate leaf (IGL); the ventral medial border was marked by the external medullary lamina (EM); the dorsal border was differentiated by the cell sparse nature of the lateral posterior nucleus (LP); the dorsal lateral border determined from visualization of the brachium of the superior colliculus (BSC). Data is displayed as a percent change in baseline (control) measure (P1, n = 7 control and n = 7 enucleates; P4, n = 6 control and n = 6 enucleates). Two-sample independent t-tests were used to compare area of nuclear gene expression in control and enucleated cases. A *P*-value of less of than 0.05 was used to establish statistical significance.

### Analysis of DAPI staining and anatomical tracing

All images were captured with a digital high resolution Zeiss Axio camera using Axiovision software (Version 4.7) coupled to a Zeiss Axio Imager Upright Microscope equipped with fluorescence at 4x magnification. DAPI stained sections were used to define thalamic nuclei boundary. Thalamic nuclear (dLGN) size was quantified in serial sections using ImageJ, with borders determined as described above. Experimenters were blind to conditions. Data is displayed as a percentage change in baseline (control) measure (n = 6 control and n = 6 enucleates). Localization and comparisons of somatosensory INCs at the S-V boundary across conditions were analyzed using an electronic micrometer measuring the distance from the cortical midline to the most medial labeled cell observed from a somatosensory cortex dye placement as was done in previous studies [21, 23] (P1, control n = 9 and enucleates n = 8; P4, control n = 11 and enucleates n = 13). Two-sample independent t-tests were conducted to statistically analyze dLGN size and INC shifts across control and enucleated brains. Values for dLGN size and INC positions are reported as the mean ± standard error. A *P*-value of less than 0.05 was chosen for statistical significance between groups.

## Results and Discussion

### Results

#### Impact of early bilateral enucleation on the dorsal thalamus of mice

**Fig 1:**
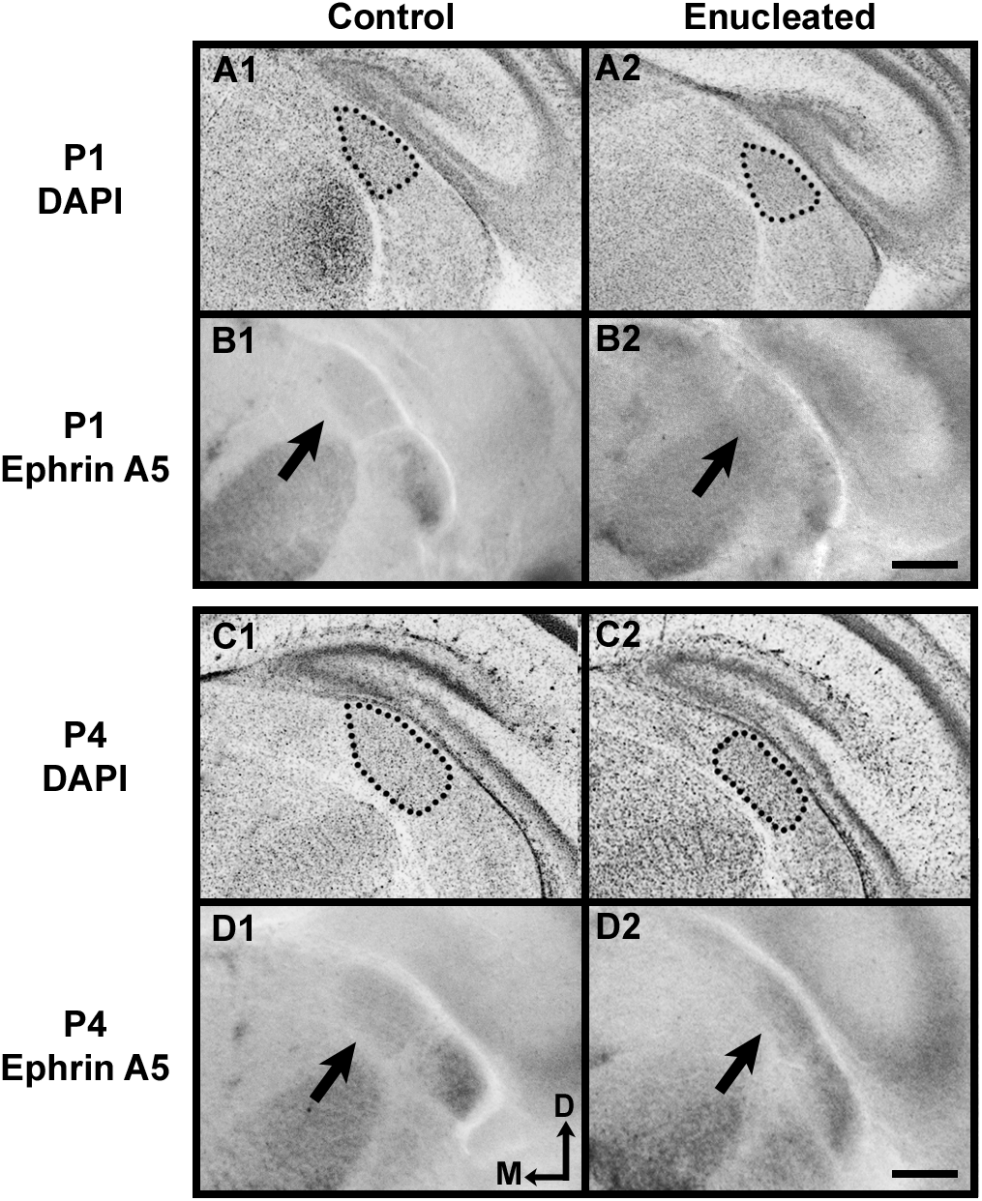
Bilateral enucleation immediately impacts dLGN size and gene expression. DAPI staining and *in situ* RNA hybridization was used to determine the size (A1-A2, C1-C2) and the distribution of transcripts for *ephrin A5* (B1-B2, D1-D2). All panels are high magnification views of the dLGN and surrounding regions of the dorsal thalamus of one hemisphere after sectioning at 100μm in the coronal plane. Dotted lines in A1-A2 and C1-C2 encircle DAPI stained dorsal thalamic nuclei (dLGN). Arrows in B1-B2 and D1-D2 indicate *ephrin A5* dLGN expression. DAPI staining 24 hours and 4 days after at-birth-enucleation resulted in a reduced dLGN size in P1 enucleated brains (A2), with total nuclei reduction being more pronounced by P4 (C2). *Ephrin A5* is expressed in a gradient in the dLGN both at P1 and P4 that is highest in ventrolateral regions (B1 and D1, respectively). Enucleation leads to a reduced area of expression of *ephrin A5* in the nucleus 1 and 4 days post enucleation at P0 (B2 and D2, respectively). Panels A2-D2 demonstrate rapid dLGN shrinkage after enucleation. All sections are oriented with dorsal (D) up and medial (M) to the left. DAPI: P1 and P4, n = 6 control and n = 6 enucleates; *Ephrin A5*: P1 n = 7 control and n = 7 enucleates, P4 n = 6 control and n = 6 enucleates. Scale bar = 200 μm.

**Fig 2:**
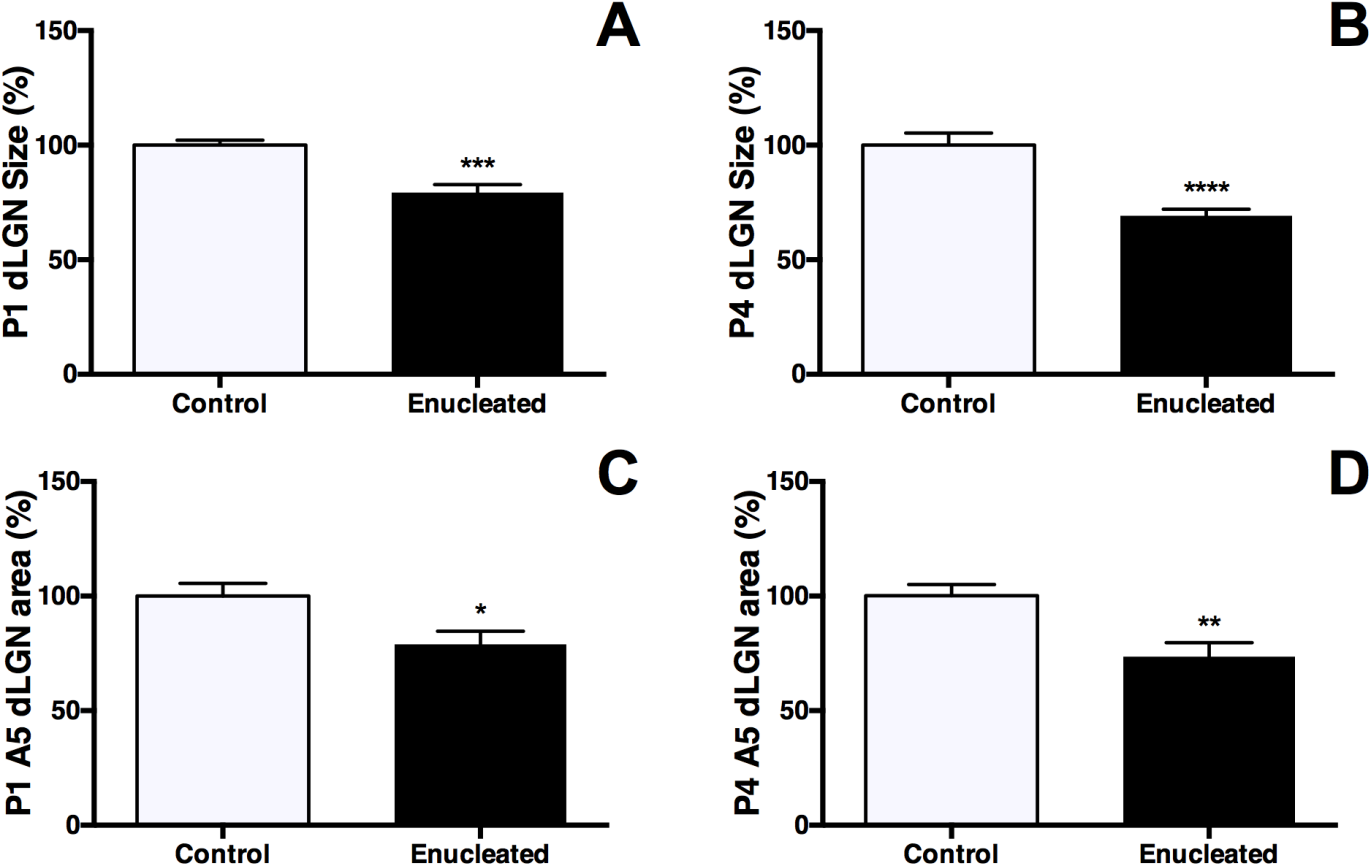
Analysis of dLGN reduction and *ephrin A5* expression in control and enucleated brains. (A) P1 cells stained with DAPI within the dLGN. Decreased nuclear size observed in enucleated cases (79.34 ± 3.462% *P* < 0.001), when compared to control animal baseline levels. (B) P4 cells stained with DAPI within the dLGN. dLGN size of enucleated animals (69.15 ± 2.938%; *P* < 0.0001) is significantly different from controls. (C) Area of *ephrin A5* expression in P1 dLGN. *Ephrin A5* expression within the dLGN of enucleated animals (78.98 ± 5.723%; *P* < 0.05) is significantly different than controls. (D) Area of *ephrin A5* expression in P4 dLGN. Percentage of *ephrin A5* expression in enucleated dLGN (73.52 ± 6.190%; *P* < 0.01) is significantly different when compared to controls. DAPI: P1 and P4, n = 6 control and n = 6 enucleates; *Ephrin A5*: P1 n = 7 control and n = 7 enucleates, P4 n = 6 control and n = 6 enucleates. All data are presented as percentage of mean control measure.

Although brain plasticity associated with sensory deprivation has been documented in several sensory systems, the precise timing of early effects of visual deprivation through bilateral enucleation has not been well described. In this study, the development of the visual sensory thalamic relay, the dLGN, was examined at two time points after early newborn bilateral enucleation: postnatal day (P) 1, and P4 (Fig 1; arrows and dotted lines highlight dLGN). DAPI, a nuclear stain, was used to assess dLGN size in control and enucleated mice 24 hours (P1) and 4 days (P4) post-enucleation. Statistical analysis of baseline corrected values revealed a rapid reduction in dLGN size present 1 day following eye removal when compared to controls (Fig 1A1 and 1A2 and Fig 2A; enucleated 79.34 ± 3.462% of control; *P* < 0.001). The observed reduction in dLGN size was more pronounced in enucleated tissue as compared to controls when assessed 4 days following early enucleation, and 8 days prior to eye opening (Figs 1C1 and 1C2 and 2B; 69.15 ± 2.938% of control; *P* < 0.0001).

**Fig 3:**
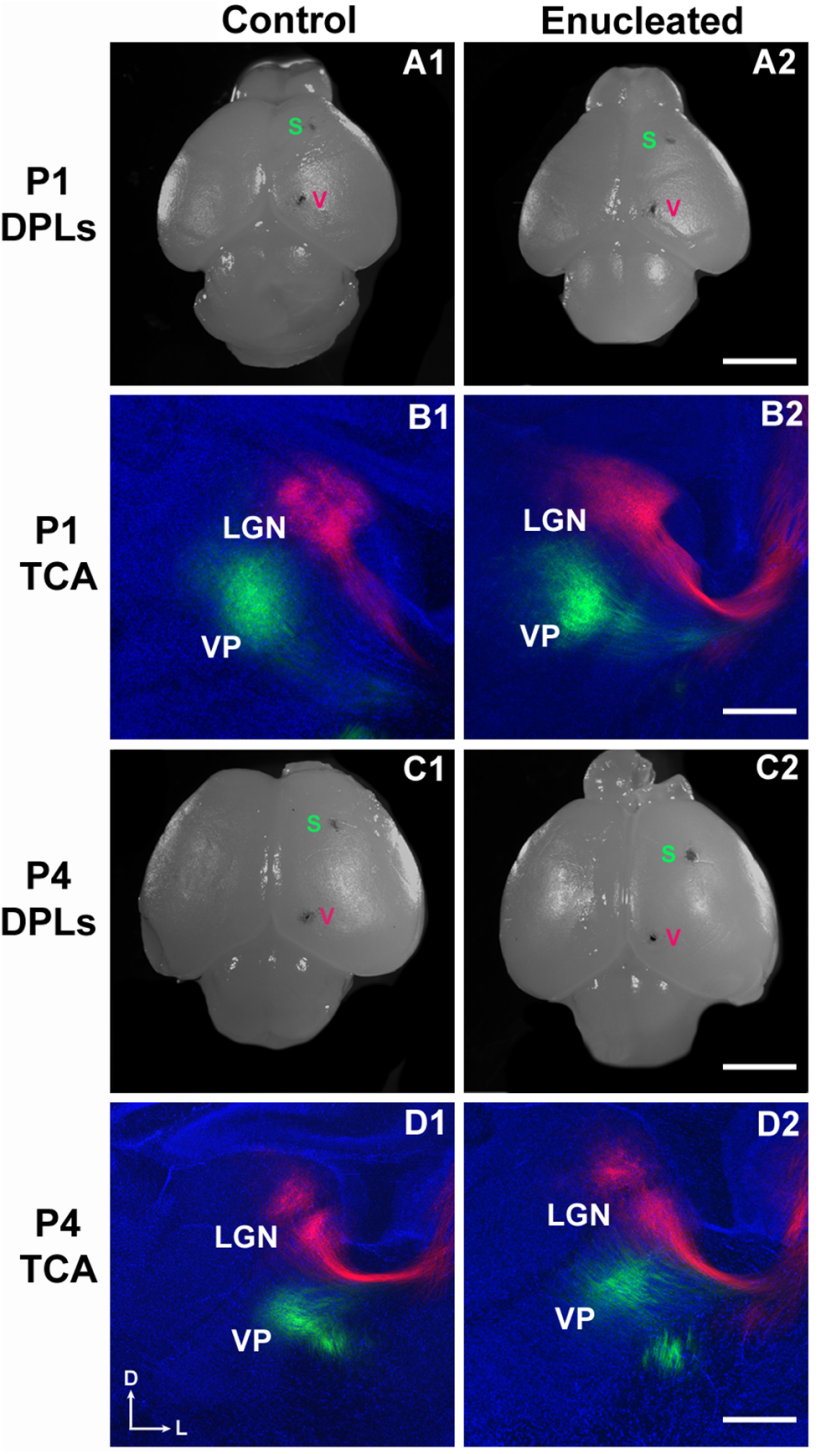
P0 enucleation does not dramatically alter thalamocortical afferent position. Dorsal views of hemisected P1, P4 control (A1 and C1, respectively) and P1, P4 enucleated (A2 and C2, respectively) brains indicating DiA putative somatosensory (S) cortex and DiI putative visual (V) cortex placement locations. High magnification views of 100μm coronal sections through the thalamus from P1, P4 control brains (B1 and D1, respectively) and P1, P4 enucleated brains (B2 and D2, respectively) are presented. Retrograded thalamocortical labeling in the dLGN and VPN was observed for both control (B1 and D1) and experimental brains (B2 and D2), which confirmed that DPLs were within the visual and somatosensory cortices. Enucleation did not appear to substantially impact the position of thalamocortical afferents despite a reduction in dLGN size. P1: n = 9 control and n = 8 enucleates; P4: n = 11 control and n = 13 enucleates. DPL: dye placement location; TCA: thalamocortical afferents; VP: ventral posterior nucleus; LGN: lateral geniculate nucleus; s: somatosensory cortex; v: visual cortex. All sections are oriented with dorsal (D) up and lateral to the left (L). Scale bar = 200 μm.

*Ephrin A5* expression in the dLGN of mice has been documented previously at birth [24] and at P10 [21]. Here we document immediate sensory driven alterations in control and bilaterally enucleated mice 1 and 4 days after bilateral enucleation. Consistent with previous studies, *ephrin A5* expression was present in both P1 and P4 control mice (Fig 1B1 and 1D1, respectively; arrows). Analysis of the area of *ephrin A5* expression in the dLGN revealed decreased levels of expression in enucleated mice when compared to wild-type mice at both P1 and P4 (Fig 1B2 and 1D2; arrows; Fig 2C 78.98 ± 5.723% of control and 2D 73.52 ± 6.190% of control; *P* < 0.05, *P* < 0.01, respectively). The area of *ephrin A5* expression remained within boundaries of the reduced nucleus at both P1 and P4. Despite a reduction in dLGN size, enucleation at birth did not appear to impact the development of thalamic afferents, observed by making dye placements in somatosensory and visual cortices (Fig 3A1-2 and 3C1-2), at P1 and P4 (compare Fig 3B1 with 3B2 and 3D1 with 3D2, respectively).

#### Early neocortical changes in gene expression after bilateral enucleation at birth

**Fig 4:**
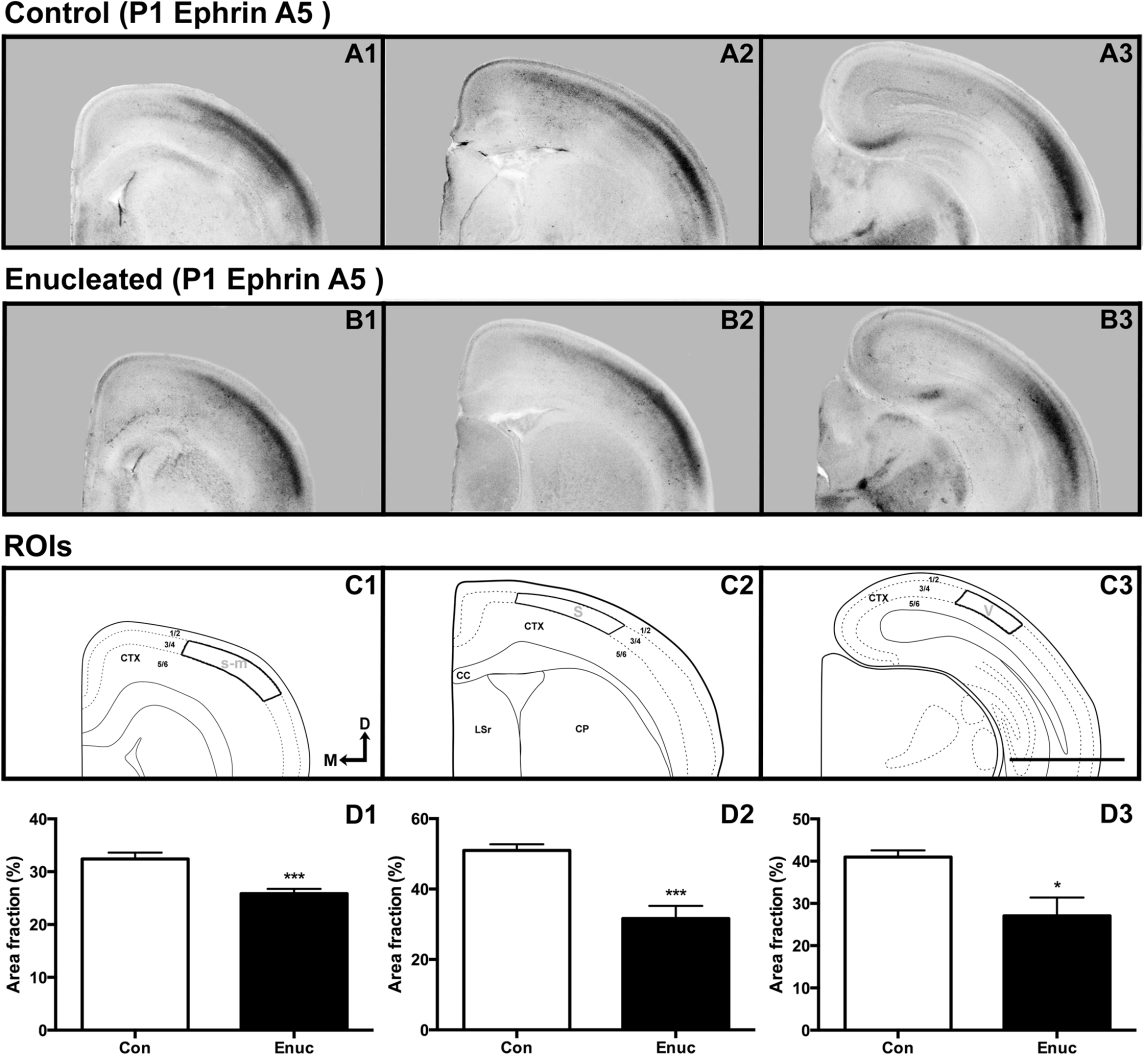
Neocortical gene expression of *ephrin A5* at P1 in control and enucleated mice. *In situ* RNA hybridization was used to determine the distribution of transcripts for *ephrin A5* at P1 in control (A1-A3) and enucleated (B1-B3) brains as labeled. All panels are low magnification views of one hemisphere after sectioning at 100μm in the coronal plane, shown in a rostral to caudal (left to right) series. Cortical *Ephrin A5* expression in control animals (A1-A3) was found predominantly in middle layers of the somatosensory-motor amalgam (A1), somatosensory (A2) and visual cortices (A3), with some extension of deep layer transcripts detected in the somatosensory and visual cortex. Expression was similar, but reduced in experimental cases (B1-B3). ROIs within the somatosensory-motor amalgam (C1), somatosensory (C2) and visual cortices (C3) all revealed significant reductions in *ephrin A5* transcript coverage (control 32.42 ± 1.199% and enucleated 25.84 ± 0.0.919%; D2, control 50.96 0078 1.741% and enucleated 31.64 ± 3.587%; D3, control 40.98 ± 1.568% and enucleated 27.07 ± 4.310%). All sections are oriented with dorsal (D) up and medial (M) to the left. S: somatosensory cortex; V: visual cortex; s-m: somatosensory-motor amalgam. Scale bar = 500μm. * = *P* < 0.05, *** = *P* < 0.001; n = 6 controls and n = 6 enucleates.

*Ephrin A5* transcripts were primarily detected in middle layers of P1 control cortex within the sensory-motor amalgam [19] (Fig 4A1), with similar patterns of expression seen in control somatosensory cortical regions (Fig 4A2). Robust levels of *ephrin A5* transcripts were also found in P1 caudal brain regions, including middle layers of putative visual cortex (Fig 4A3). In the enucleated animals, however, *ephrin A5* transcripts were detected at lower levels in the sensory-motor amalgam (compare Fig 4A1 with 4B1). Further analysis of transcript density in sensory-motor cortex ROI (Fig 4C1) revealed significantly lower levels of transcripts in enucleated brains when compared to controls (Fig 4D1; control 32.42 ± 1.199% and enucleated 25.84 ± 0.0.919%; *P* < 0.01). Decreased *ephrin A5* expression was also detected in medial somatosensory cortical areas of P1 enucleated brains when compared to controls (compare Fig 4A2 with 4B2). Analysis of gene expression levels in P1 control and enucleated medial somatosensory ROI (Fig 4C2) showed significantly reduced percentage of area above threshold in enucleated brains when compared to control cases (Fig 4D2; control 50.96 ± 1.741% and enucleated 31.64 ± 3.587%; *P* < 0.01). *Ephrin A5* percentage of area above threshold in P1 visual cortex ROI (compare Fig 4A3 with 4B3; Fig 4C3) was significantly reduced in enucleated cases when compared with controls (Fig 4D3; control 40.98 ± 1.568% and enucleated 27.07 ± 4.310%; *P* < 0.05).

**Fig 5:**
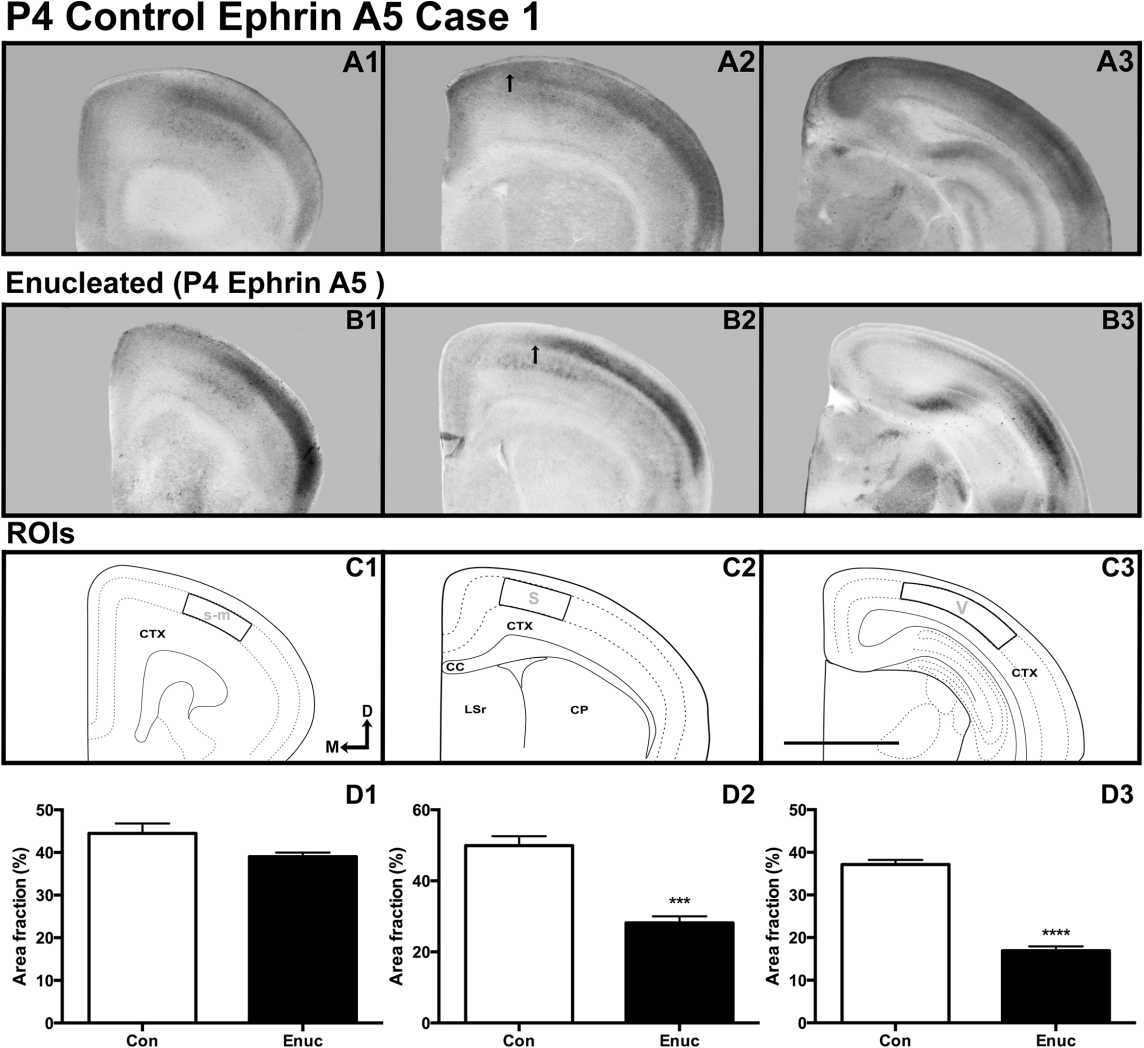
Neocortical gene expression of *ephrin A5* at P4 in control and enucleated mice. All panels are high magnification views of 100μm coronal sections of P4 brain hemispheres following *in situ* RNA hybridization with probes directed against *ephrin A5* (A1-B3). *Ephrin A5* expression in the somatosensory-motor amalgam (A1-B1; ROI in C1) exhibited no significant difference between control and experimental animals (control 44.50 ± 2.308% and enucleated 39.02 ± 0.978%). *Ephrin A5* transcripts within control somatosensory cortex extend further medially (compare arrows in A2 and B2) into superficial layers when compared to enucleated cases (D2, ROI in C2; control 49.94 ± 2.654% and enucleated 28.18 ± 1.869%; *P* < 0.001). ROIs within visual cortex (C3) revealed significant reductions of *ephrin A5* expression in enucleated animals when compared to controls (D3; control 37.14 ± 1.076%, enucleated 16.94 ± 0.996%; *P* < 0.0001). S: somatosensory cortex; V: visual cortex; s-m: somatosensory-motor amalgam. All sections oriented dorsal (D) up and medial (M) to the left. Scale bar = 500μm. N = 6 controls and n = 6 enucleates.

At P4, expression of *ephrin A5* was observed at very low levels to absent levels in deep layers throughout the cortex, but present at varying intensity in middle layers of both control and enucleated animals. Robust *ephrin A5* expression was observed in sensory-motor regions of control brains (Fig 5A1), with very similar patterns of expression levels detected in enucleated sensory-motor cortex (compare Fig 5A1 with 5B1). Further analysis of P4 sensory-motor amalgam ROI (Fig 5C1) in both control and enucleated brains revealed a P value that indicates a trend towards a significant difference in transcript density within this region (Fig 5D1; control 44.50 ± 2.308% and enucleated 39.02 ± 0.978%; *P* = 0.0605). Transcripts in control somatosensory cortex were detected in middle layers. Transcripts were also detected in middle layers of enucleated somatosensory cortex, although the expression border was lateral to that of control cases (compare arrows in Fig 5A2 and 5B2). Analysis of a P4 control and enucleated cortical ROI (Fig 5C2) revealed decreased area above threshold in enucleated tissue, when compared to controls (Fig 5D2; control 49.94 ± 2.654% and enucleated 28.18 ± 1.869%; *P* < 0.001). A significant difference in *ephrin A5* area above threshold between enucleated and control visual cortices was detected (compare Fig 5A3 with 5B3), with enucleated brains expressing significantly lower levels of gene expression in the visual cortex ROI (Fig 5C3) when compared to control animals (Fig 5D3; control 37.14 ± 1.076% and enucleated 16.94 ± 0.996%; *P* < 0.0001).

#### Bilateral enucleation at birth does not alter intraneocortical connectivity in early postnatal life

**Fig 6:**
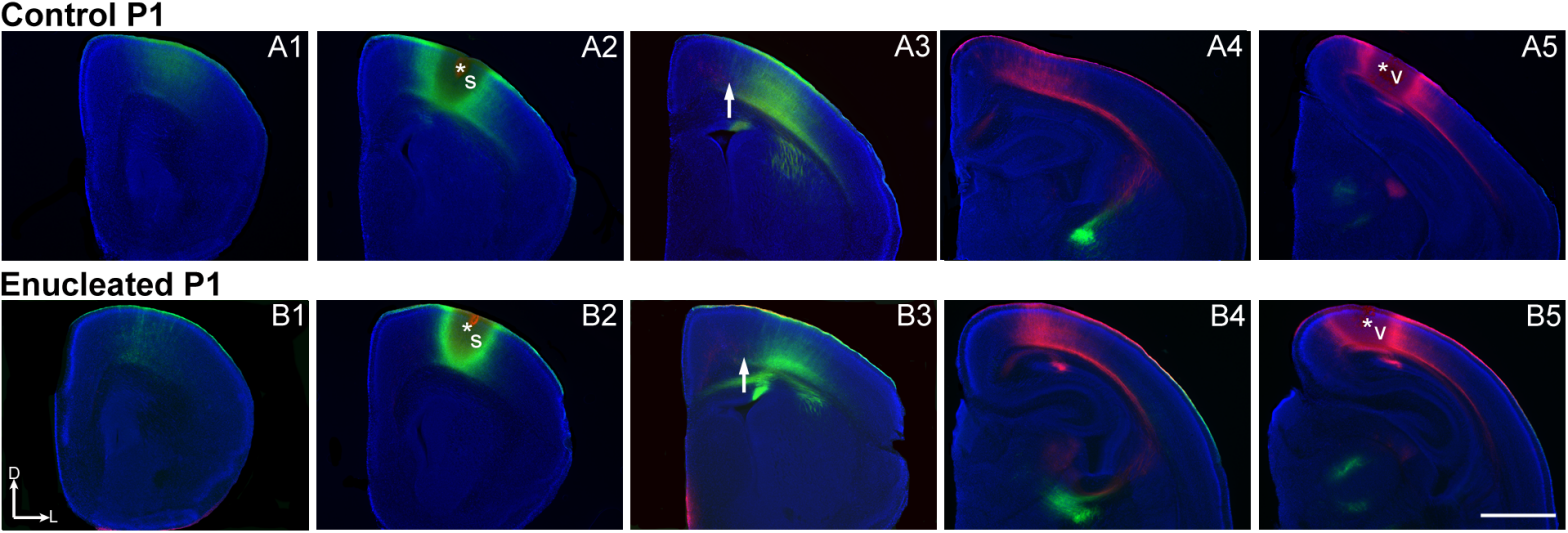
Bilateral enucleation does not alter INCs in P1 brains. Rostral to caudal series of 100μm coronal sections of P1 hemispheres following placement of DiA or DiI crystals in putative visual or somatosensory cortices to label INCs in control (A1-A5) and enucleated brains (B1-B5). In representative control and enucleated brains, retrogradely labeled cells from a DPL in the parietal somatosensory cortex (A1-A4, and B1-B4, respectively) do not overlap with cells or axons labeled by an occipital visual cortex DPL (A3 and B3, respectively). The arrows in A3 and B3 indicate the region of non-overlap between the labeling fields of the two areas, which represents the presumptive S-V areal boundary at P1. Dorsal (D) is up and lateral (L) is to the right. Scale bar = 1mm. Asterisks indicate DPL. DPL: dye placement location; INC: ipsilateral intraneocortical connection; P: postnatal day; s: somatosensory areas; v: visual cortex; S-V: somatosensory-visual. N = 9 controls and n = 8 enucleates.

In order to determine the effects of bilateral enucleation at birth on INCs early in development, DiI and DiA were placed in postmortem brains of P1 and P4 control and enucleated mice. Cortical area boundaries have been previously determined using specific arrangement of INCs within different sensory areas [16,18–19]. INC tracing from a DiA (green) DPL in the parietal somatosensory cortex reveal retrogradely labeled cells observed in positions rostral and caudal to the DPL (Fig 6A1–6A3 and 6B1–6B3; green, DPL starred in 6A2 and 6B2) in both control and enucleated brains. DiI visual cortex DPL (DPL starred in Fig 6A5 and 6B5) resulted in INCs rostral (Fig 6A4 and 6B4) and caudal relative to the DPL (data not shown), with deep cortical axonal labeling (Fig 6A4 and 6B4) in both control and enucleated neocortex. There did not appear to be any major differences in the targeting of INCs for these dye placements in control and enucleated mice.

**Fig 7:**
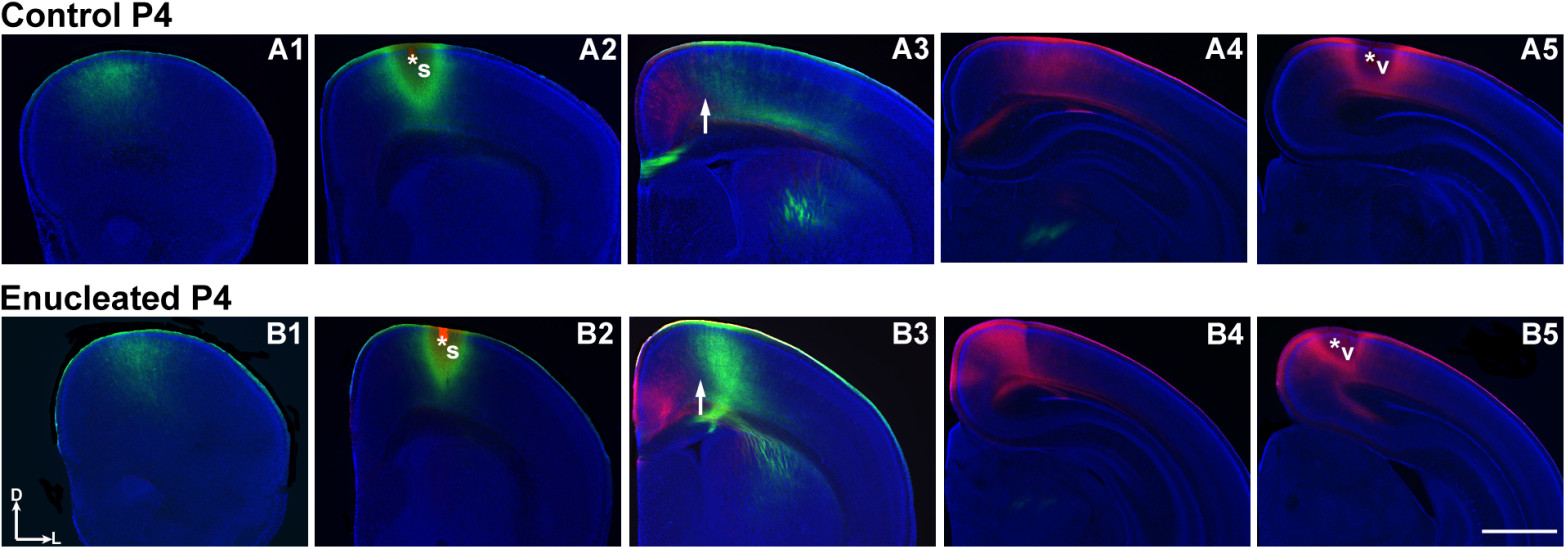
No alterations in INCs four days after early enucleation. Rostral to caudal series of 100 μm coronal sections of P4 hemispheres following placement of DiA or DiI crystals in putative visual or somatosensory cortices to label INCs in control (A1-A5) and enucleated (B1-B5) brains. In control and enucleated animals, there are retrogradely labeled cells resulting from a DPL in the parietal somatosensory cortex both rostral and caudal to the DPL (green labeling in A1-A4 and B1-B4, respectively), with no overlapping of cells or axons labeled by an occipital visual cortex DPL (red labeling in A3 and B3). The arrows in A3 and B3 indicate the region of non-overlap between the labeling fields of the two areas, which are representative of medial S-V areal boundary at P4. Dorsal (D) is up and lateral (L) is to the right. Scale bar = 1mm. Asterisks indicate DPL. DPL: dye placement location; INC: ipsilateral intraneocortical connection; P: postnatal day; s: somatosensory areas; v: visual cortex; S-V: somatosensory-visual. N = 11 controls and n = 13 enucleates.

**Fig 8:**
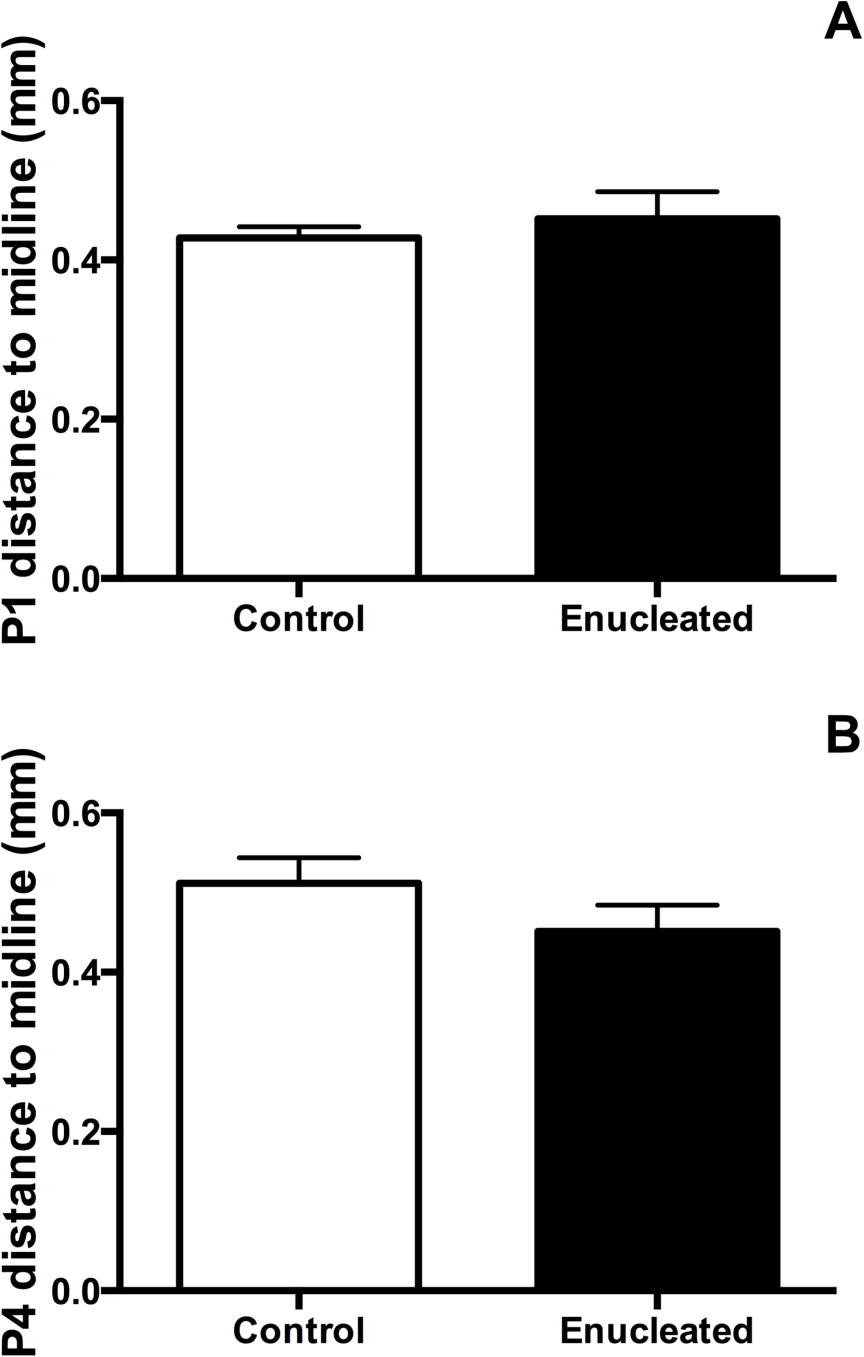
Analysis of dye labeling in control and enucleated brains. (A) Distance to midline in P1 control and enucleated mice. No significant difference in somatosensory border distance from midline was detected between groups (control, 0.43 ± 0.01 mm, n = 9; enucleated, 0.45 ± 0.03 mm, n = 8; P = 0.50). (B) Distance to midline in P4 control and enucleated mice. Somatosensory border distance from midline was did not differ significantly between groups (control, 0.51 ± 0.03 mm, n = 11; enucleated, 0.45 ± 0.03 mm, n = 13; P = 0.20).

In P4 control and enucleated brains, DiA placement in the parietal somatosensory cortex retrogradely labeled cells in locations rostral and caudal (Fig 7A1–7A3 and 7B1–7B3; green) to the DPL (DPL starred in Fig 7A2 and 7B2). Similarly, when DiI was placed in visual cortex of P4 control and enucleated mice, INCs rostral (Fig 7A3 and 7A4 and 7B3 and 7B4, respectively) and caudal (data not shown) relative to the DPL were labeled (DPL starred in Fig 7A5 and 7B5). Enucleation-induced changes in INC position, as defined by distance of dye labeled cells to midline, were not observed at P1 or P4 (Fig 8A; control, 0.43 ± 0.01 mm and enucleated, 0.45 ± 0.03 mm; *P* = 0.50; Fig 8B; control, 0.51 ± 0.03 mm; enucleated, 0.45 ± 0.03 mm; *P* = 0.20), as present in P10 blind mice [21]. This suggests that early bilateral enucleation rapidly alters gene expression in the thalamus and cortex and these changes are later followed by alteration to cortical connections.

In summary, by removing both eyes at birth, we observed immediate changes in subcortical and cortical regions. Correlated with a reduction in dLGN size, we demonstrate the reduction of a well-characterized guidance molecule, *ephrin A5*. This reduced *Ephrin A5* expression in the dLGN occurs alongside reduced cortical gene expression in enucleated brains when compared to controls. These changes in gene expression occur before shifts in intraneocortical connectivity are observed, as we did not observe alterations in dye tracing at P1 or P4.

## Discussion

There is ample data to support the role that genes play in early brain patterning *and* the very important role of sensory input in the formation of sensory thalamic nuclei and neocortical areas [16,18–19,21]. These oppositional data sets have established a foundation for understanding mechanisms underlying sensory patterning in the brain. Most visual neuroscientists today would argue that both genes and sensory experience interact in some way to create a segregated and highly functional visual system. However, understanding how these contributions interact is not a simple process. One way to approach this is to use sensory deprivation models where the developing brain is deprived of sensory experience. Most early sensory deprivation studies report subcortical and cortical alternations later in life [25], however there is a paucity of data describing how changes in sensory input may impact gene expression. In the present report, we extend our previous study, which described the impact of newborn bilateral enucleation on neocortical gene expression at P10. In that study, we found that within 10 days of blindness, the intraneocortical connections of mouse primary visual cortex were altered in a location that was co-registered with an ectopic pattern of *ephrin A5* expression [21]. Although a novel and exciting finding, the methodology did not allow us to determine whether the blindness induced a change in gene expression *first*, followed by a re-routing of cortical connections or *vice versa*. The study described here analyzes the impact of newborn bilateral enucleation, or visual sensory deprivation, on the brain within the first postnatal week in order to determine which feature of cortical organization was altered first as a result of the deprivation. Based on previous work from our laboratory, we hypothesized that an abrupt change in sensory input via bilateral enucleation would induce a rapid change in gene expression leading to a later alteration of intraneocortical connectivity.

Rapid effects of bilateral enucleation on dLGN A high impact result from our study is the very rapid change in the size of dorsal LGN, its reduced gene expression as well as the reduced *ephrin A5* expression in cortex as early as 24 hours post-blindness. Specifically, DAPI staining and *in situ* RNA hybridization revealed a dorsomedial reduction in dLGN size and *ephrin A5* expression in both P1 and P4 enucleates (Figs 1 and 2) without a significant change to thalamocortical connectivity (Fig 3). Consistent with past studies indicating a reduction in dLGN post-enucleation [26–32], our results demonstrate that the dLGN was still present however its size was rapidly reduced and the expression of *ephrin A5* in the nucleus was decreased, with thalamocortical afferent patterning remaining unchanged. As thalamocortical connections are established by birth in mice [33], it is not surprising that a short-term sensory deprivation did not significantly impact this anatomy.

Reduced dorsal LGN size resulting from altered sensory input has been previously associated with loss of neurons and glia, and decreased soma size [28,34]. Our results are consistent with previous studies which demonstrate that bilateral enucleation in early postnatal mice causes the dLGN to lose more than half its volume due to a decrease in the size of neurons and in the number of neurons and glial cells in the dLGN [28]. In addition, primates enucleated prenatally also show more than a 50% reduction in dLGN volume, which can be attributed to a decrease in the number of cells [35–36]. In the current study, however, we observed a rapid change in nuclear size *and* gene expression; this is a novel finding as previous studies examined long-term effects of sensory deprivation on the LGN. It is unclear whether removing all spontaneous retinal activity in our early enucleation model led to the observed rapid reduction in dLGN size due to cell death and concurrent loss in neurons and glia, *or* that enucleation caused a rapid change in *ephrin A5* expression in the dLGN, which led to subsequent reduction in nuclear size. Further experiments, perhaps with *ephrin A5* knockout mice, would be needed to address this question.

*Ephrin A5* signaling has been shown to be critical in the regulation of normal retinogeniculate and thalamocortical topography [24,37]. For example, dLGN projections to the visual cortex was disrupted in both ephrin-A2/A3/A5 triple knockout mice and mice experimentally manipulated to misexpress ephrin-A2 or–A5 in V1 [32]. More notably, Pfeiffenberger and colleagues [24] showed disrupted retinogeniculate mapping when inhibiting correlated spontaneous neural activity in ephrin-A2/A3/A5 triple knockout mutants. Severe disruptions in the visual cortex was also observed when disrupting cholinergic retinal waves in ephrin double knockout (ephrin-A2/A5) mice, thus highlighting the importance of neural activity and ephrin-As acting together to control retinogeniculate and retinocortical patterning in normal development [14,24]. Taken together, our data suggests that retinal activity early in life regulates proper thalamic *ephrin A5* expression and anatomy. Thus, entire removal of retinal spontaneous wave activity at birth tends to alter thalamic *ephrin A5* gene expression as early as 24 hours post-sensory deprivation, with alterations becoming more pronounced by P4.

Effects of short-term enucleation on neocortical gene expression In addition to changes in LGN size and gene expression, early bilateral enucleation altered *ephrin A5* expression in neocortex as early as 1 day after enucleation at P0. This change in gene expression within multiple, putative sensory and motor areas persisted to P4. How spontaneous retinal activity relates to *ephrin A5* expression in multiple areas within cortex is not known; however, complete cessation of retinal input to the developing cortex initiated a rapid change in the position of *ephrin A5* expression across cortex only 1 and 4 days after enucleation. Several genes involved in partitioning the cortex into distinct sensory and motor areas are expressed in gradients across the cortical sheet. When enucleation and subsequent visual deprivation occurs, we suggest that areas of high expression at the poles of the cortex shift, impacting the gradients of other genes expressed across the sheet. For example, *Pax6* and *Emx2* are expressed early on in counter gradients, with *Pax6* expressed high rostral to low caudal and *Emx2* expressed high caudal to low rostral. When *Emx2* (strongly expressed caudally in putative visual cortex) is knocked out in the mouse, the expression gradient of *Pax6* shifts caudally [38]. Thus, we suggest that visual deprivation alters expression of genes in caudal cortex, which then impacts the expression of genes throughout other cortical regions including somatosensory and motor cortex.

It is not surprising that we see a shift in gene expression levels in our early enucleation experiments, as the developing mammalian brain is subject to anatomical and functional reorganization following peripheral alternations during development [39–41]. Additionally, mice with suppressed retinal wave activity during the period of cortical map formation show imprecise retinocortical mapping and disruption in visual cortex development [32]. Supporting this notion, previous electrophysiological studies by Krubitzer and colleagues have demonstrated cross-modal neocortical plasticity, whereby removal of retinal spontaneous activity in early development resulted in a clear expansion of auditory and somatosensory cortical areas into regions of cortex that would normally be subjected to visual innervation in adulthood [42–43]. Similarly, studies examining visual cortex development in visually deprived cats have demonstrated activation of auditory neurons in visual cortical regions [44]. Thus, both contraction and expansion of sensory cortex arealization has been observed in adult mammalian neocortex with loss or enhanced use of peripheral receptors [45]. Consistent with animal model studies assessing neocortical development following disruption of peripheral activity, multiple studies in congenitally blind individuals have also shown the remarkable capacity of the neocortex to acclimate to sensory loss. Specifically, a shorter detection time for auditory discrimination tasks and faster language processing has been observed in congenitally blind humans [46–47]. Neuroimaging studies of blind individuals have also shown activation of regions normally involved in visual processing during auditory localization tasks and Braille reading [48–50]. Taken together, these data, coupled with our findings of altered *ephrin A5* gene expression, emphasize the contribution of retinal waves to both cellular and molecular mechanisms of the developing cortex and provide grounds to speculate the role of *ephrin A5* in mediating cross-modal neocortical plasticity as a result of modifying patterns of peripheral activity.

The alterations in *ephrin A5* expression in P1 and P4 neocortex, including the lateral positioning of expression in P4, observed in enucleated mice may not be a ‘shift’ in expression, or a shift in area position, but rather a delay in developmental processes. This notion stems from an observation that cortical expression in P1 and P4 enucleated brains is similar to pre- and perinatal expression in the same regions of neocortex in wild-type mice [18–19]. More notably, the recovery of significantly reduced *ephrin A5* expression in P1 sensory-motor cortex at P4 seen here, strongly supports the idea of delayed expression of key developmental genes following peripheral modifications (Figs 4 and 5). An interruption in cortical development after abrupt sensory deprivation is not out of the question, as early visual deprivation studies have shown a delay in ocular dominance critical period plasticity [51–55]. Our findings are consistent with studies suggesting that dark rearing has profound effects on genetic profiles regulated by visual sensory input. In a series of experiments examining cortical development, Majdan and Shatz [56] reported changes in gene regulation, including a decrease in gene expression, resulting from a decrease in visual activity via dark rearing. These authors suggested that molecular processes and experience work in tandem to establish mature circuits and proper critical periods in the developing neocortex. Thus, studies showing profound effects of age-specific gene regulation after a brief dark rearing period early in development, coupled with a decreased *ephrin A5* expression observed in our P1 and P4 enucleated brains, may illustrate a potential mechanism underlying prolonged critical periods in neocortical development. In order to determine if the short-term plasticity actually caused a shift in borders or area position within sensory and motor cortex, multi-unit electrophysiology would be needed to determine functionality of the region. Unfortunately, this is not feasible at these early ages. However, tracing data presented here linking regions of interest within the cortex to appropriate thalamic nuclei suggest that the areas have not changed their position within cortex.

Effects of short-term bilateral enucleation on ipsilateral INCs Ipsilateral somatosensory and visual cortex INC development is well characterized in the first week of life in CD-1 mice [18,19]. In the current study, consistent with our thalamocortical connectivity observations, day-of-birth enucleation resulted in no change in INC patterning within the first few days of life, (Figs 6, 7 and 8) despite a published alteration in connectivity in the same model at P10 [21].

Early developmental processes have been shown to involve and rely heavily on endogenous spontaneous activity from the retina and sensory input transmitted through the eyelid [32,57–59]. Our data in P10 mouse following newborn bilateral enucleation [21] supports this notion where a change in both gene expression and intraneocortical connectivity was observed. Enucleation-induced changes in cortical connectivity occur in the second postnatal week after gene expression is modified.

## Conclusions

In this study we used a sensory deprivation model to determine whether deprivation leads to a change in gene expression followed by a remodeling of intraneocortical connections, or *vice versa*. Previous data led us to hypothesize that acute visual deprivation achieved through bilateral enucleation would first generate a change in cortical gene expression followed by remodeling of sensory INCs in cortex. Our data supports this hypothesis as a rapid change in cortical *ephrin A5* expression is detected just one day after bilateral enucleation in the newborn mouse neocortex with normal connections prevailing until P4. Our initial study demonstrated that by P10, cortical *ephrin A5* expression and somatosensory INCs were altered, leading us to conclude that the alteration of sensory INCs in our blindness model occurs between P5 and P10, after a change in gene expression. A rapid effect of bilateral enucleation was also seen in the thalamus. Just 24 hours after enucleation, we observed that the dLGN was significantly reduced in size following removal of all spontaneous retinal activity. By studying the effects of significant perturbations of sensory system development on the anatomy of sensory nuclei, important developmental genes and INCs, we have begun to characterize the immediate effects of enucleation and the relationship between vision loss, dLGN anatomy and cortical *ephrin A5* expression.

We suggest that input from the retina is critical for precise gene expression in and development of thalamic nuclei and cortical sensory areas, as we observed modifications in *ephrin A5* expression in these regions only 1 day after sensory deprivation. These rapid effects observed both in the thalamus and cortex within the first postnatal week offer novel insights into potential interactions of molecular processes and peripheral sensory input, highlighting the importance of retinal activity in the first few days of life for proper establishment of precise temporal gene expression in sensory areas. These data suggest that cortical gene expression plays a role in the development of proper intraneocortical connectivity, providing further evidence for the interaction of the two features of brain development first discussed by Huffman [16]. Further studies in *ephrin A5* mutant mice would need to be conducted to better interpret the role of this gene and activity-dependent mechanisms in the formation and maintenance of INCs in the first few days and weeks of post-natal development.
