# Correction: Rapid Changes in Cortical and Subcortical Brain Regions after Early Bilateral Enucleation in the Mouse

**DOI:** 10.1371/journal.pone.0142461

**Published:** 2015-11-04

**Authors:** Olga O. Kozanian, Charles W. Abbott, Kelly J. Huffman

Figs 3 and 5 are incorrect. The authors have provided the corrected versions here.

**Fig 3:**
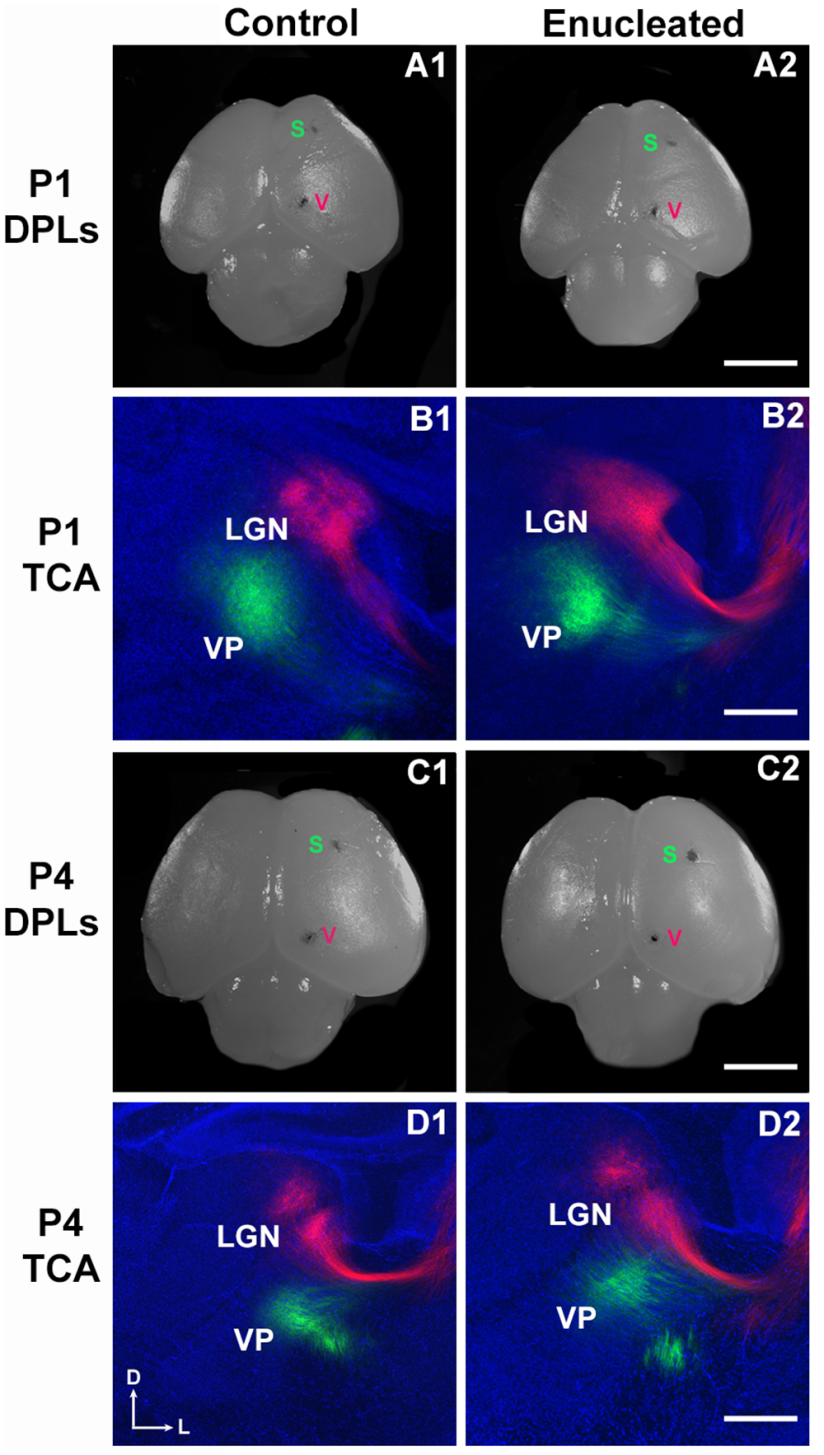
P0 enucleation does not dramatically alter thalamocortical afferent position. Dorsal views of hemisected P1, P4 control (A1 and C1, respectively) and P1, P4 enucleated (A2 and C2, respectively) brains indicating DiA putative somatosensory (S) cortex and DiI putative visual (V) cortex placement locations. High magnification views of 100μm coronal sections through the thalamus from P1, P4 control brains (B1 and D1, respectively) and P1, P4 enucleated brains (B2 and D2, respectively) are presented. Retrograded thalamocortical labeling in the dLGN and VPN was observed for both control (B1 and D1) and experimental brains (B2 and D2), which confirmed that DPLs were within the visual and somatosensory cortices. Enucleation did not appear to substantially impact the position of thalamocortical afferents despite a reduction in dLGN size. P1: n = 9 control and n = 8 enucleates; P4: n = 11 control and n = 13 enucleates. DPL: dye placement location; TCA: thalamocortical afferents; VP: ventral posterior nucleus; LGN: lateral geniculate nucleus; s: somatosensory cortex; v: visual cortex. All sections are oriented with dorsal (D) up and lateral to the left (L). Scale bar = 200 μm.

**Fig 5:**
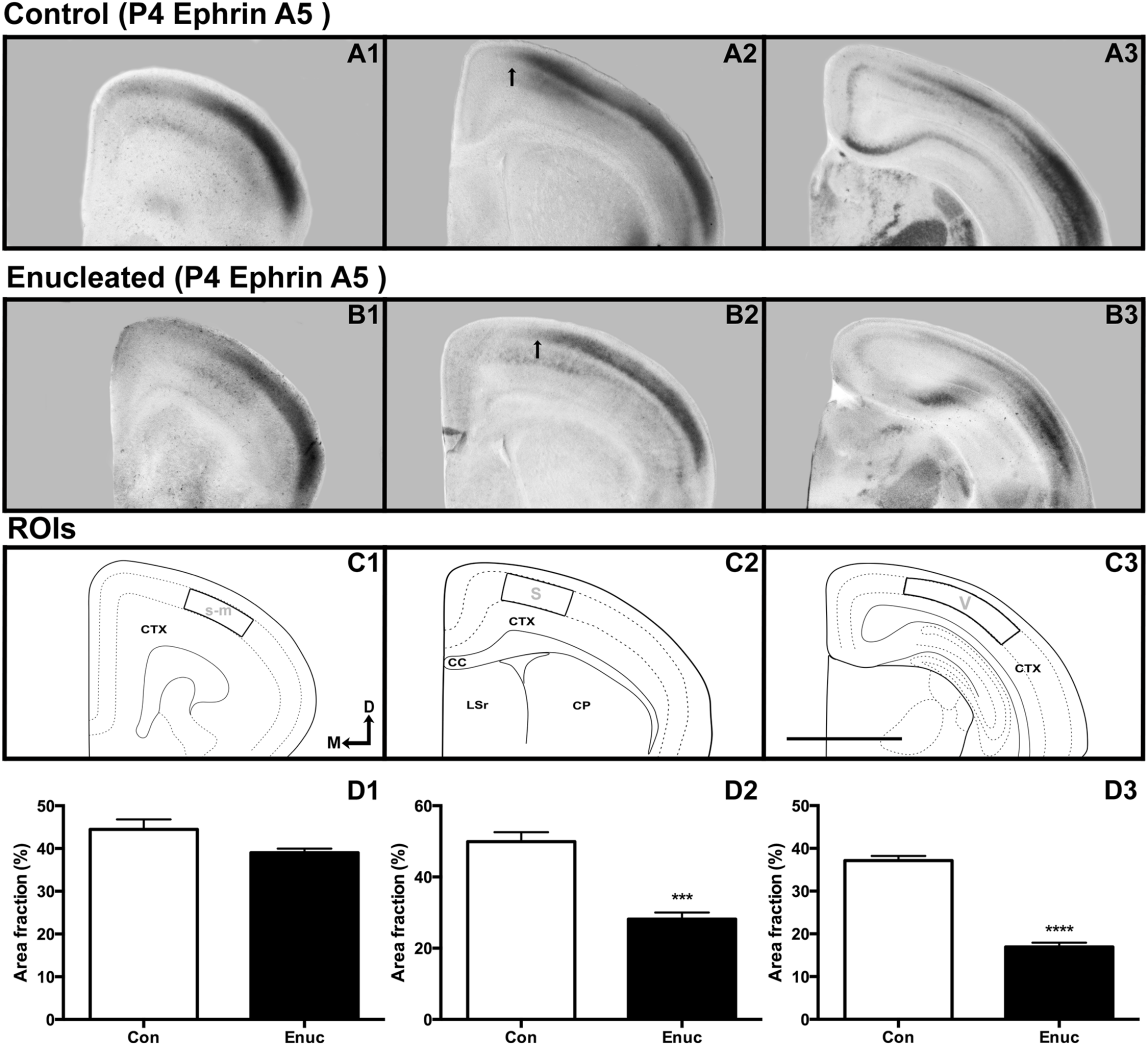
Neocortical gene expression of ephrin A5 at P4 in control and enucleated mice. All panels are high magnification views of 100μm coronal sections of P4 brain hemispheres following in situ RNA hybridization with probes directed against ephrin A5 (A1-B3). Ephrin A5 expression in the somatosensory-motor amalgam (A1-B1; ROI in C1) exhibited no significant difference between control and experimental animals (control 44.50 ± 2.308% and enucleated 39.02 ± 0.978%). Ephrin A5 transcripts within control somatosensory cortex extend further medially (compare arrows in A2 and B2) into superficial layers when compared to enucleated cases (D2, ROI in C2; control 49.94 ± 2.654% and enucleated 28.18 ± 1.869%; P < 0.001). ROIs within visual cortex (C3) revealed significant reductions of ephrin A5 expression in enucleated animals when compared to controls (D3; control 37.14 ± 1.076%, enucleated 16.94 ± 0.996%; P < 0.0001). S: somatosensory cortex; V: visual cortex; s-m: somatosensory-motor amalgam. All sections oriented dorsal (D) up and medial (M) to the left. Scale bar = 500μm. N = 6 controls and n = 6 enucleates.
